# Anterior gland focal cryoablation: proof-of-concept primary prostate cancer treatment in select men with localized anterior cancers detected by multi-parametric magnetic resonance imaging

**DOI:** 10.1186/s12894-019-0562-5

**Published:** 2019-12-05

**Authors:** Christina Sze, Efrat Tsivian, Kae Jack Tay, Ariel A. Schulman, Leah G. Davis, Rajan T. Gupta, Thomas J. Polascik

**Affiliations:** 1 Urologic Surgery, Duke University Medical Center and the Duke Cancer Institute, Durham, NC USA; 2 Division of Urology, Department of Surgery, Duke University Medical Center, Box 2804, Yellow Zone, Durham, NC 27710 USA; 3 Singapore General Hospital, Singapore, Singapore; 4 Department of Radiology, Duke University Medical Center, Durham, NC USA

**Keywords:** Focal cryoablation, Anterior gland, Prostate cancer, Erectile function, Continence

## Abstract

**Background:**

Due to their location away from the nerve bundles, anterior prostate cancers (APC) represent a rational target for image-guided cryoablation. This report describes the feasibility and short-term outcomes of anterior focal cryosurgery.

**Methods:**

A retrospective review between 2012 and 2016 of patients with clinically localized APC treated with anterior gland cryoablation was performed. Descriptive statistics were used to report: age, PSA, prostate volume, prostate cancer grade group (PGG), median time to follow-up, and changes in functional status measured with the International Prostate Symptom Score (IPSS) and the International Index of Erectile Function (IIEF-5) score.

**Results:**

A total of 17 patients underwent anterior focal cryoablation with a median follow-up of 15 months. Median age and PSA at diagnosis were 67 years and 8.7 ng/mL. Pre-operative PGG1 was identified in 12 (71%) men and PGG2 in 5 (29%) men. Median (IQR) lesion volume was 2 mL(0.86, 3.1). Preoperative median IIEF-5 and IPSS scores were 19.5 and 5, and decreased to 19 and 4, post-operatively. All patients remained continent with no change in sexual function. All post-procedure targeted biopsies of the treated cancers were negative.

**Conclusion:**

Our pilot study demonstrates the feasibility of treating APCs with image-guided targeted focal cryoablation as a good balance between short-term oncologic control and near complete preservation of genitourinary function. Further follow-up is necessary to examine the potential benefits long-term.

## Background

Prostate Cancer (PCa) is the second leading cause of cancer-related deaths among men in the United States with the American Cancer Society estimating 164,699 new cases and 29,430 deaths in 2018 [1]. Conventional methods for treating PCa, such as radical prostatectomy or radiation therapy, although effective, often cause inadvertent but significant damage to surrounding structures leading to erectile dysfunction (ED), incontinence, or bowel irritation to a variable degree [2]. Many men report complete social withdraw from sexual contact and intimacy leading to relationship discord, frustration, and depression [3].

Advances in techniques and associated technologies, such as nerve-sparing focal therapy (FT), have proven to be effective in treating patients with localized prostate cancer in the short and intermediate term, and when applied in a targeted manner provide excellent preservation of sexual and urinary function [4, 5]. One such therapy, focal cryotherapy, uses small needles to deliver lethal cold energy that causes direct cellular damage, tissue necrosis, and resultant apoptosis through the formation of ice crystals and other mechanisms [4]. Reported potency rates from this procedure can range from 60 to 90% and depend on preoperative erectile function and tumor location in relationship to the neurovascular bundles (NVB) [4]. Even with these advancements, a large disparity in post-surgical potency exists, making it challenging to predict sexual function outcomes.

Anterior prostate cancer (APC) accounts for 10–30% of PCa [6]**.** Due to its location, APCs represent a good therapeutic target for focal cryoablation. APCs are located far from the neurovascular bundles (NVBs), allowing for adequate surgical margins while preserving nerve integrity. However, their location is also a diagnostic blind spot and is often missed with systematic biopsies and the digital rectal examination [7]. Consequently, patients frequently present with higher PSA levels, larger tumor volumes, and often have histories of multiple biopsies [8]. The development and widespread adoption of multi-parametric MRI (mpMRI) for clinical use have improved the earlier detection and localization of these previously occult tumors [9].

This study describes the feasibility and short-term outcomes of anterior focal cryoablation as a new technique applied to a mpMRI-characterized disease entity.

## Methods

### Patient selection

After receiving institutional review board approval, a retrospective review was performed between 2012 to 2016 of patients who were treated with anterior gland cryoablation for clinically localized cancer. All patients had a prostate mpMRI. If a suspicious lesion was described on mpMRI, patients underwent image-targeted mpMRI-TRUS fusion biopsy to confirm the malignant nature of the lesion. At least 2 cores were obtained from the target lesion followed by a systematic biopsy sampling of 6–12 cores, depending on the prostate volume. Patients without lesions seen on mpMRI (*n* = 1), discordance between mpMRI and previous biopsy (*n* = 2), or due to patient preference (n = 2) underwent 3D transperineal template-guided prostate mapping biopsy (TTMB). Our 3D TTMB technique has been previously described [10]. Erectile and urinary functional outcomes were assessed by self-reported standardized instruments using the International Index of Erectile Function (IIEF-5) and the International Prostate Symptom Score (IPSS), respectively. No patient had received any other primary treatment for their prostate cancer. An smaller cohort was expected given the novelty of the technique. Cryoablation was performed by a single provider.

### Multi-parametric MRI technique

Our mpMRI technique is similar as described previously in our work [11]. Briefly, mpMRI scans were obtained utilizing 3.0 Tesla scanners (Signa HDx, GE Healthcare, Waukesha, WI and Skyra, Siemens Healthcare, Erlangen, Germany) with a single-channel endorectal coil as well as multichannel surface coils. The imaging protocol includes multiplanar fast spin-echo T2-weighted imaging (T2WI), diffusion-weighted imaging (DWI) using multiple *b* values and calculation of apparent diffusion coefficient (ADC) maps. Dynamic contrast-enhanced imaging sequences were obtained after administration of a weight-based dose of extracellular MRI contrast agent with a 4- to 5-s temporal resolution for 5–6 min. All image interpretation lesion and segmentation was carried out on by a third party analysis software (DynaCAD, Invivo Corp.,Gainesville, FL) by a single board-certified, fellowship-trained radiologist with 5 years of experience in interpreting prostate mpMRI.

### Surgical technique

**Fig. 1:**
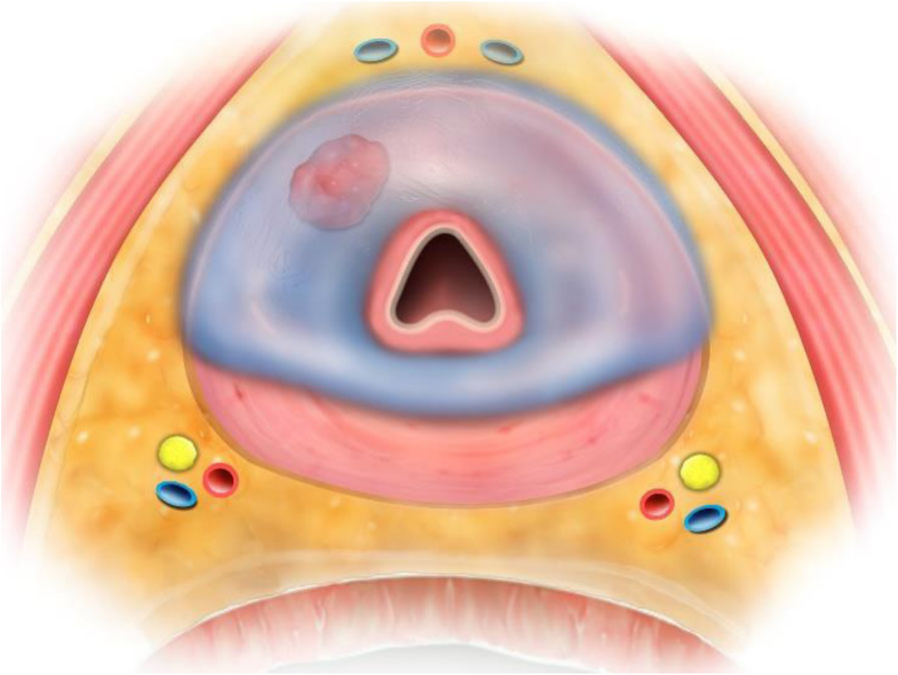
Transaxial view of the prostate illustrating anterior cryoablation template. Ice, depicted in transparent blue, can be run posteriorly all the way to the peripheral zone leaving the neurovascular bundles [yellow/red/blue circles] untouched. Tumor depicted anteriorly in red, urethra in center of schematic

Our anterior gland focal cryotherapy technique has been previously published [12]. Briefly, freezing the entire prostate generally involves the placement of three horizontal rows of 2–4 cryoprobes from the anterior to posterior gland. Each probe is placed within 2 cm of one another beginning with the most anterior row. Anterior gland ablation is defined as using up to only the first two anteriorly positioned horizontal rows of the standard probe template. The traditional posterior row of cryoprobes are not placed, thereby allowing a bilateral nerve sparing procedure. Temperature thermocouples are positioned to monitor the temperature of the following structures: urethral sphincter, Denonvillier’s fascia, and the NVBs on both sides (Fig. 1). Cystoscopy is then performed to ensure no probe violates the urethra. Two freeze-thaw cycles are performed. At no time is the ice edge enabled to come close to the NVBs nor is the temperature around those structures allowed to go below 35C. This is an outpatient procedure and the patient is discharged with a short-term urethral catheter, an alpha-blocker and belladonna/opium suppositories.

### Follow-up

The 2015 International Consultation on Urological Disease consensus on follow-up after focal therapy was used as a reference [13]. Patients are advised to follow-up 3 months post-operatively and then every 6 months for the next 5 years. At every visit, PSA, DRE, continence status, IIEF-5 and IPSS scores were recorded. At approximately 12 months post-operatively, patients underwent a mpMRI with targeted biopsy of the treated area, and any new suspicious lesions. A systematic biopsy (6–12 cores) of the untreated area was also performed for purposes of active surveillance due to the multifocal nature of PCa. Those patients without lesions on mpMRI or negative biopsies are to be re-evaluated with biopsy and mpMRI imaging at 5 years post-operatively. MpMRI surveillance continues annually otherwise through 5 years.

### Statistical analysis

Descriptive statistics have been generated using median and interquartile range for continuous data (limited sample size) and counts and percentages for binary, ordinal and categorical endpoints. Clinically significant changes in IIEF-5 scores prior to and post procedure was defined by a difference of 4 points [14]. All analyses were performed using R 3.3.3 software.

## Results

Between 2012 to 2016, 17 patients with complete follow-up were identified with APC and elected treatment with anterior gland prostate cryotherapy. The median serum PSA, and prostate volume at cryoablation were 8.7 ng/ml (6.7–11.76), and 35 cc (24–47.7), respectively. At the time of ablation, the median number of biopsy sessions a patient had undergone was 3 [2, 3]. Pre-ablation mpMRI identified anterior gland lesions in 16/17 (94%) of subjects with a median tumor volume of 2 cc (0.86, 3.1) (Table 1). To confirm the mpMRI detected lesion, the majority (71%) underwent a pre-treatment mpMRI-TRUS fusion biopsy of whom 25% chose to have only the mpMRI detected lesions sampled while 75% had a sextant biopsy in addition. A total of 5 (29%) patients elected TTMB incorporating targeted biopsy of the lesion along with detailed mapping comprising at least 50 cores. No patients had both types of biopsies (e.g. transrectal fusion plus TTMB) prior to cryoablation. One patient was found to have anterior prostate cancer by TTMB alone. A 3-D volume of the tumor was calculated based on mapped location of the cancer in X-Y-Z coordinates along with needle core cancer length. PGG1 disease was present in 12 (71%) patients while the remaining patients (29%) had PGG2 disease. Those with PGG1 or PGG2 disease had similar median lesion volumes on mpMRI, 2 cc (0.74, 2.94) and 2.04 cc (0.98, 4.5), respectively.

**Table 1:** Patient and disease characteristics pre-ablation

Characteristic	Value
PSA (ng/ml), median (IQR)	8.7 (6.7, 11.76)
TRUS Prostate Volume (cc), median (IQR)	35 (24.3, 47.7)
No. prior biopsy sessions^†^, median (IQR)	3 (2,3)
MRI lesion volume (mL), median (IQR):	
Overall	2 (0.86, 3.1)
PGG 1	2 (0.74, 2.94)
PGG 2	2.04 (0.98, 4.5)
Biopsy type^‡^:	
Fusion, No. (%)	12 (71)
TTMB, No. (%)	5 (29)
No. cores^‡^, mean (range)	24.8 (1, 68)
No. positive cores ^b^ (%):	
1	11 (64.7)
2	4 (23.5)
4	2 (11.8)
PGG, No. (%)	
1	12 (71)
2	5 (29)

*PGG* prognostic grade group, *TTMB* transperineal template-guided prostate mapping biopsy

^†^Includes most recent biopsy performed at Duke

^‡^Most recent biopsy performed at Duke prior to cryoablation

**Fig. 2:**
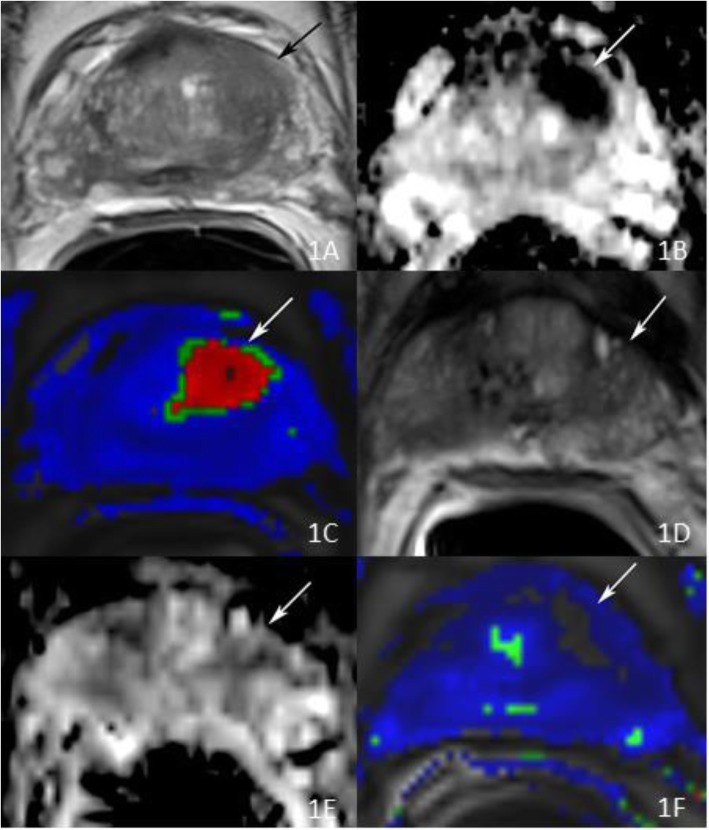
**a** Axial T2-weighted (T2W) image reveals ill-defined decreased T2 signal at the level of the left anterior transition zone at the level of the base (arrow). **b** Axial apparent diffusion coefficient (ADC) map demonstrates markedly restricted diffusion in this region (arrow). **c** Colored perfusion map created using post-processing software from dynamic contrast-enhanced MRI (DCE-MRI) acquisition demonstrates suspicious perfusion kinetics for prostate cancer (arrow), corresponding to the findings seen on T2W and DWI. This lesion was scored as a PI-RADS 4 and patient underwent MRI-US fusion biopsy which revealed Gleason 3 + 3 = 6 prostate cancer at the targeted area. Based on these findings, patient elected to undergo anterior focal cryoablation of this lesion. **d**,**e**,**f** Axial T2W image, ADC map and colored perfusion map created using post-processing software from DCE-MRI acquisition in the post-ablation setting reveals ablation defect with no suspicious findings in the area of treatment and specifically, no abnormal perfusion kinetics. Patient’s PSA continued to decrease with a nadir at 0.6 ng/mL confirming successful targeted anterior cryoablation

Post-ablation PSA data was available for 15/17 (88%) patients. The median PSA nadir and time to nadir were 0.82 ng/ml (0.55, 1.75) and 4 months (3, 7.5), respectively (Table 2). Follow-up mpMRI was performed in 9/17 (53%) patients at a median time of 14 months [13, 15] after ablation. mpMRI did not identify any lesions in all patients. Post ablation biopsy was performed in 10/17 (59%) patients. Biopsies of all ablated areas were negative for cancer (Fig. 2).

**Table 2:** Post-ablation imaging, biochemical and pathological data

Characteristic	Value
Follow-up (mos.), median (IQR)	15 (13,17)
PSA nadir (ng/ml), median (IQR)	0.82 (0.55, 1.75)
Time to nadir (mos.), median (IQR)	4 (3, 7.5)
Time to MRI (mos.), median (IQR)	14 (13, 15)
No. positive biopsy (%):	
PGG 1	2 (100)

*PGG* prognostic grade group

However, random surveillance biopsy in 2 patients revealed 1 mm or less of clinically insignificant or PGG1 disease in an untreated area of the prostate (Table 3). These two patients had PSA elevation at 6 and 13 months and PSA nadir of 2 ng/ml and 3.7 ng/ml respectively. IPSS scores were available for all 17 subjects. Median (IQR) pre- and 12-month post-ablation IPSS scores were 5 (2, 0) and 4 (2,4), respectively with a median difference between pre- and post-ablation scores of − 2 (− 3,0). IIEF-5 scores were available for 16/17 patients. Median pre- and 12-month post-ablation IIEF-5 scores were 19.5 [16, 17] and 19 [14, 18], respectively with a median difference between pre- and post-ablation scores of − 1 (− 3.25, 0). Eight subjects (47%) had a pre-operative IPSS score < 5 and 13 subjects had an IPSS score < 5 (76%) at > 12 months post-operatively. Three subjects (19%) had a pre-operative IIEF-5 score > 21 and 3 (19%) subjects had an IIEF-5 score > 21 at > 12 months post-operatively. Median time from surgical intervention to follow-up IIEF-5 and IPSS scores were 15 months. No Clavien-Dindo complications or incontinence requiring absorbent pads were reported.

**Table 3:** Details of two recurrences detected on follow-up

Patient	Ablated anterior gland location	Pre ablation MRI lesion volume (cc)	Pos biopsy location Location		PGG	CCL (mm)	% CCL
1	Left medial apex TZ	3	Left lateral apex & Left lateral mid		1	Core 1: < 1 Core 2: < 1	Core 1: 5% Core 2: 2%
2	Left medial apex TZ	0.73	left lateral apex		1	1	7.7%

*PGG* prognostic grade group, *CCL* core cancer length, *TZ* anterior transition zone, *Pos* positive

## Discussion

This proof-of-concept study describes the feasibility of a new image-guided focal therapy application for appropriate candidates with APC aimed to achieve good oncological outcomes with preserved continence and erectile function [15]. Treatment of early PCa diagnosed in younger or sexually active patients deserves consideration as post-treatment sexual dysfunction can often lead to psychosocial consequences. Patient selection is one of the hallmarks of any FT application. Patients with PGG1 disease opted for anterior ablation due to large median lesion size of 2 mL, high suspicion score on mpMRI (lesions were either PIRADS 4 or 5) despite PGG1 by targeted biopsy, or not ideal candidates for active surveillance but considered over-treated if radical therapy was selected. High-volume Gleason pattern 3 has an increased risk of harboring higher grade cancer and has a one-in-ten chance of capsular invasion [19].

The anatomical and biological behavior of APCs are unique, and its definition varies. Villers defined the anterior borders of the prostate as the region of parenchyma at least 2.1 cm anterior to the posterior capsule that represents an area the transrectal biopsy needle characteristically fails to reach [20]. Anatomically, this is a portion of the prostate anterior to the urethra which includes areas of McNeal’s transition zone, the anterior fibromuscular stroma and the anterior horns of the peripheral zone [16]. APCs can be derived from any of these tissue origins making it heterogenous as a group. Our study emphasizes the significance of APCs from a therapeutic perspective in that the defined target area is both located anteriorly and sufficiently away from the NVBs, and the treatment of which would foster a true bilateral nerve-preserving ablation without potentially sacrificing oncological outcomes in well-selected men.

Recently, a case series of patients with low- and intermediate-risk APCs treated with robotic partial prostatectomy demonstrated high continence rates of 100% and potency rates of 83% at 3 months post-operatively [20]. Procedures were completed without intraoperative complications however perioperative complications included anastomotic leak (12%), urinary tract infection (6%) and transient intestinal ileus (6%). These are risks not usually encountered when utilizing FT especially since targeted ablation is commonly performed as an outpatient procedure given its low perioperative morbidity. In cryotherapy, preventive measures such as use of a warming catheter are employed to prevent injury to the external striated sphincter and urethra when treating APCs. Thus, in anterior cryoablation, indirect injury to these structures are rarely seen. Additionally, temperature thermocouples are placed to monitor any potential changes to surrounding tissue, particularly the NVBs. Therefore, focal ablation is a viable option with minimal to no significant morbidity for treating APCs.

Although the concept of APC is not new, the role of mpMRI in detecting and staging APCs is one that has gained recent interest. The conventional tool to diagnose PCa, relying on random TRUS-guided biopsies (10–12 cores), is more adept at detecting posterior PCa. On average, detection of APCs requires more repeat biopsies compared to posteriorly located PCa and require inserting the biopsy needle deeper into the parenchyma [21]. In our series, patients typically have a history of at least 2 rounds of biopsies prior to APC detection. mpMRI-TRUS guided fusion biopsy increases the detection of APC and have been shown to significantly detect more APCs than TRUS-guided biopsy especially in those who have histories of previous negative TRUS-guided biopsies [17, 18]. This superior performance is due to the superior anatomic resolution of of T2 weighted-images (T2WI) combined with functional techniques such as apparent diffusion coefficient (ADC) mapping of mpMRI [22]. Given the increased incorporation of mpMRI into clinical decision-making, it is expected APCs will be increasingly detected in the future, possibly enabling more men to be considered candidates for anterior gland ablation. mpMRI is an additional diagnostic modality that has value in the initial clinical staging nomograms and may lead to fewer cores being taken and potentially be cost effective [23].

In this series, the median pre-operative IPSS scores decreased by 2 at 12 months post-operatively suggesting an improvement in urinary symptoms although not a significant change in this cohort as these men were not very symptomatic at onset. However, 5 additional men had a 1-year post-ablation IPSS < 5, suggesting further improvement in urinary function post-treatment as measured by this self-reported QOL instrument. The use of anterior gland FT seems to have little adverse impact on urinary function which is consistent with other reports on FT [24]. The very high rate of preserving urinary continence appears unique to FT in general and anterior gland ablation in particular. Previous studies have shown urinary incontinence rates ranging from 0 to 3.6% after FT [25]. Regarding erectile function, we allowed patients to self-report their scores before and after anterior gland cryoablation utilizing the validated IIEF-5 instrument. The median difference in IIEF-5 between pre- and post-ablation scores was − 1 following anterior gland ablation suggesting minimal change in sexual performance. Conceptually this is logical as the energy field is located far from the NVBs.

In terms of oncological outcomes, patients were free of clinically significant PCa on follow-up biopsy in the short term in those undergoing evaluation. Prostates were subjected to intense scrutiny with mpMRI, targeted biopsy of the ablated tumor and random, conventional biopsy of the untreated parenchyma on active surveillance. Two subjects demonstrated biopsy-proven low volume, ≤ 1 mm in each core, PGG1 disease in the untreated region at the apex. The apex is not uncommonly an area where recurrences or cancer persistence may occur post ablation largely due to conservative freezing of the area to avoid potential damage to the external sphincter [26]. mpMRI is the recommended imaging modality after FT because of its high negative predicative value (NPV) and sensitivity to detect clinically significant cancer, particularly high volume or high grade disease [27]. Previously, Radtke et al. showed that there is value in the use of systematic biopsy with the image-targeted biopsy as the combination of the two identified more clinically significant PCa than mpMRI alone [28]. The combination of extended biopsies along with targeted fusion biopsy augments the NPV of mpMRI to maximize the likelihood to detect persistent cancer or new occult tumors as PCa is typically multifocal.

We recognize certain limitations of this study. First, this is a retrospective extended case series with a smaller sample size therefore only descriptive analyses were completed. It is also worth mentioning that though each patient underwent anterior cryoablation, the procedures were customized according to the location of the tumor(s) and shape of the prostate. However, customization reflects a real-world application of FT whereby treatments should be individualized according to tumor location, size and focality. This series also consisted of a group of men having a relatively small mean prostate volume that has been shown to impact oncologic outcomes as larger prostate provides a surgeon a greater margin of error when performing FT [29]. Additionally, the study relies on patient reported measures to assess urogenital function using validated instruments that has its own merits and limitations regarding patient-reported data. This series was restricted to a short timeframe of median follow-up time 15 months. However, although this is a very short time frame to judge oncologic outcomes, it is of sufficient time to make determinations regarding functional outcomes. Due to the excellent functional profile that FT offers for maintaining potency and continence, many patients often feel they are back to baseline health relatively quickly. The disadvantage of such rapid recovery is that the patient may no longer feel the need to follow-up medically, therefore making acquisition of longer-term follow-up data even more challenging to accumulate. Of note, no peri-operative complications such as urinary tract infections or uncontrolled bleeding were recorded.

## Conclusions

This extended cohort series describes the feasibility and short-term outcomes of anterior gland focal cryoablation as a new technique applied to a mpMRI-characterized disease entity, the APC. These findings provide clinicians and patients with data that could inform expectations regarding functional outcomes and short-term cancer control following this procedure. Ultimately, patients must weigh the relative value of cancer control and preservation of genitourinary function. Additional follow-up is needed to evaluate long-term outcomes of FT for APCs and better define those patients who may benefit most from this technique.

## Data Availability

**\**All data generated or analyzed during this study are included in this published article [and its supplementary information files].

## Abbreviations

APC: Anterior prostate cancers
FT: Focal therapy
IIEF-5: International Index of Erectile Function
IPSS: International Prostate Symptom Score
mpMRI: Multiparametric magnetic resonance imaging
NVBs: neurovascular bundles
PCa: Prostate cancer
PGG: prostate cancer grade group
TRUS: Transrectal ultrasound
TTMB: Transperineal template-guided prostate mapping biopsy
